# Supplementary material for: Content-rich biological network constructed by mining PubMed abstracts
Source: BMC Bioinformatics. 2004 Oct 8;5:147. doi: 10.1186/1471-2105-5-147 (PMC528731; doi:10.1186/1471-2105-5-147)
Supplement: Additional File 2 — The original results of the above study (non-essential files are deleted to keep the file size under the limit set by BMC bioinformatics). [file 1471-2105-5-147-S2.bz2 › chilibotAdditionalFile2/dip05/496ID10187774E10659/html/ACVR2_ACVRL1.html]

 


 **ACVR2** and **ACVRL1** 
  
Found 62 abstracts in PubMed, retrieved 05.  
 

 What does Google say? 
 PDF only 
| .edu only 

---

**Interactive relationship** (e.g. stimulation, inhibition, etc)

**Neutral relationship**- Activin receptor  [ **ACVR2** ]  like kinase 1 ALK 1  [ **ACVRL1** ] , the gene mutated in HHT  [ **ACVRL1** ]  type 2 HHT2  [ **ACVRL1** ] , is a serine threonine kinase receptor type I of the TGF beta superfamily, specifically expressed on endothelial cells.  Ref: 12700602 Eur J Hum Genet, 2003
- HHT  [ **ACVRL1** ]  type 2 is caused by loss of function mutations in activin receptor  [ **ACVR2** ]  like kinase 1  **ACVRL1**  or ALK1  [ **ACVRL1** ] .  Ref: 12588795 Hum Mol Genet, 2003
- Hereditary hemorrhagic telangiectasia HHT  [ **ACVRL1** ]  is a vascular disorder in humans which has been mapped to two genes, endoglin and activin receptor  [ **ACVR2** ]  like kinase 1 ALK 1  [ **ACVRL1** ]  both of which mediate signaling by transforming growth factor beta ligands in vascular endothelial cells.  Ref: 12667943 Cardiovasc Res, 2003

**Non-interactive relationship** (e.g. studied together, co-existance, homology, etc.)

- Recent studies have identified mutations of the bone morphogenetic protein receptor 2 BMPR2 gene and the activin receptor  [ **ACVR2** ]  like kinase 1 ALK1  [ **ACVRL1** ]  gene, that affect the transforming growth factor beta TGF beta receptor superfamily, a group of transmembrane signaling molecules with serine threonine kinase activity that are involved in the regulation of cell growth.  Ref: 12624796 Z Rheumatol, 2003
- A preliminary investigation was performed to exclude association between the predisposition and certain candidate genes including KDR kinase insert domain receptor, TEK TEK tyrosine kinase endothelial,  **ACVRL1**  activin receptor  [ **ACVR2** ]  like kinase 1, ENG endoglin and FLT4 fms like tyrosine kinase 4.  Ref: 12752572 Clin Genet, 2003
